# Gastric adenocarcinoma and periodontal disease: A systematic review and meta-analysis^☆^

**DOI:** 10.1016/j.clinsp.2023.100321

**Published:** 2024-02-01

**Authors:** Francisco José Nunes Aguiar, Fabrício dos Santos Menezes, Marcela de Araújo Fagundes, Gisele Aparecida Fernandes, Fabio de Abreu Alves, João Goncalves Filho, Maria Paula Curado

**Affiliations:** a Post Graduation Program A. C. Camargo Cancer Center, São Paulo, Brazil; b Department of Health Education, Universidade Federal do Sergipe, Aracaju, SE, Brazil; c Nucleus of Epidemiology and Statistics in Cancer, A. C. Camargo Cancer Center, São Paulo, Brazil; d Head of Service, Oral Medicine Department, A. C. Camargo Cancer Center, São Paulo, Brazil; e Stomatology Department, Faculdade de Odontologia, Universidade São Paulo, São Paulo, SP, Brazil; f Head and Neck Surgery and Otorhinolaryngology Department, A.C. Camargo Cancer Center, São Paulo, Brazil

**Keywords:** Gastric neoplasia, Periodontal disease, Periodontitis, Epidemiology

## Abstract

**Background:**

The oral cavity is a link between of external environment with gastrointestinal tract. Studies are controversial on the presence of Periodontal Disease (PD) and its association with Gastric Adenocarcinoma (GAC).

**Methods:**

The authors performed a systematic review and meta-analysis to verify the association between PD and GAC. Six electronic databases were evaluated between 1961 and 2022. Titles and abstracts were reviewed independently according to the eligibility criteria, assessing full texts of selected studies. The quality of the included research was verified using the Newcastle-Ottawa Scale for case-control and cohort studies. Statistical analyses were performed based on fixed and/or random effects models to calculate the summarized Relative Risk (RR) and its 95 % Confidence Interval (95 % CI).

**Results:**

There were 639 studies, of which nine articles were included (3 case-controls and 6 cohorts). Overall, the authors identified 1,253 cases of GAC 2,501 controls in case-control studies, and 1,631 patients with GAC enrolled in cohort studies. Patients presenting PD increased the risk of developing GAC by 17 % (RR=1.17; 95 % CI 1.03‒1.32), which remained regardless of the diagnostic method for PD, i.e., clinical examination (RR = 1.19; 95 % CI 1.14‒1.24) and self-report (RR = 1.34; 95 % CI 1.06‒1.69). Moreover, Asian patients (RR=1.17; 95 % CI 1.00‒1.36) with PD had a higher risk of having GAC than American and European patients (RR = 1.18; 95 % CI 0.84‒1.66).

**Conclusions:**

The presence of PD the risk of GAC suggesting that its infectious-inflammatory process of PD may be related to GAC development. Further investigations on the oral-gastric microbiota and its role in the carcinogenesis of gastric cancer should be carried out, and the screening of patients with potential risk for GAC should be considered in the clinical practice of dentists.

## Highlights

• Patients presenting periodontal disease increased the risk of developing gastric adenocarcinoma by 17 %.
• The association remained regardless of the diagnostic method for periodontal disease, i.e., clinical examination and self-report.
• Moreover, Asian patients with periodontal disease had a higher risk of having gastric adenocarcinoma than American and European patients.

## Introduction

In 2020, Gastric Cancer (GC) was the malignancy that had more than one million new cases and about 770,000 deaths worldwide. Being it is fifth most frequent cancer and the fourth cause of cancer death in the world.^1^ Its risk factors already known, in addition to infection by *Helicobacter pylori* (*H. pylori*), are obesity, excessive intake of salt and meat, low consumption of fruits and vegetables, smoking, alcoholism, and low socioeconomic status, they are associated with gastric carcinoma and other malignancies.^2^^,^^3^

Periodontal diseases affect up to 50 % of the world's population and rank sixth among the most prevalent pathologies worldwide.^4^ Alterations in oral dysbioses change the oral microbiome, which may lead to oral pathologies such as Periodontal Disease (PD). Gingivitis and periodontitis are the most common forms of PD. This disease has different clinical signs of inflammation limited to the gum (gingivitis), while periodontitis results in progressive destruction of the periodontal ligament and alveolar bone, forming pouch, gingival retraction, or both^5^^,^^6^ and tooth loss is considered a result a significant increase in periodontal diseases in individuals over 40 years of age.^7^ Therefore, there is some evidence about that dysbiosis occurring in the oral cavity, such as periodontal disease, is a trigger for cancer, such as gastric adenocarcinoma.^8^^,^^9^

But epidemiological evidence on the association of PD and GAC remains limited and controversial, where some studies suggest positive associations, reporting that the infectious-inflammatory process of PD is capable of initiating inflammation mediators and microorganisms that may initiate the carcinogenesis^10^, ^11^, ^12^, ^13^, ^14^, ^15^ however some studies had null results, mainly due to, lack of pattern about data on exposure.^16^, ^17^, ^18^ This study aimed to conduct a systematic review with meta-analysis to investigate the an association between PD and GAC.

## Materials and methods

This project was registered on the International Prospective Register of Systematic Reviews ‒ PROSPERO platform on January 18, 2021, under registration code CRD42021221317.

### Literature search

This systematic review was conducted and reported in accordance with PRISMA guidelines.^19^ Articles were identified through searches limited to the English language on the PubMed, Embase, Web of Science, Scopus, Lilacs and Opengrey databases. The search strategy was based on different terms for each database (Table 1).

**Table 1:** Search strategy.

DATABASE	WEBSITE	SEARCH STRATEGY	TOTAL
Medline/PUBMED via National Library of Medicine	http://www.ncbi.nlm.nih.gov/pubmed	(((((((Periodontal Diseases [mh]) OR (Periodontal Diseases)) OR (Periodontal Diseases [tiab])) OR (periodontitis)) OR (periodont*)) OR (((((gingivitis [mh]) OR (gingivitis)) OR (gingivitis [tiab])) OR (gingivitis)) OR (gingivitis*))) OR ((((((tooth loss [mh]) OR (tooth loss)) OR (tooth loss [tiab])) OR (tooth loss)) OR (tooth loss*)))) AND (((((((Stomach Neoplasms [mesh]) OR (Stomach Neoplasms)) OR (stomach cancer)) OR (gastric adenocarcinoma)) OR (adenocarcinoma [tiab])) OR (gastric cancer)))	353
Embase	www.embase.com	('periodontal disease' OR gingivitis OR periodontitis OR 'tooth loss') AND 'gastric cancer'	74
Scopus	www.scopus.com	(TITLE-ABS-KEY (periodontal AND disease) OR TITLE-ABS-KEY (periodontitis) OR TITLE-ABS-KEY (gingivitis) OR TITLE-ABS-KEY (tooth AND loss) AND TITLE-ABS-KEY (gastric AND cancer))	137
Lilacs	https://lilacs.bvsalud.org/	"(periodontal diseases OR periodontitis OR gingivitis OR tooth loss) AND (stomach neoplasms OR gastric cancer)"	0
Web of science	www.webofknowledge.com	TI=(cancer) AND TS=(periodontal* diseases OR gingivitis OR periodontitis OR tooth* loss AND gastric cancer*) AND AB=(stomach cancer OR gastric cancer AND periodontal disease OR gingivitis OR periodontitis OR tooth loss) AND TI=(stomach cancer OR gastric cancer AND periodontal disease OR gingivitis OR periodontitis OR tooth loss)	75
OpenGrey	http://www.opengrey.eu	"periodontal disease" OR “gingivitis” OR “periodontitis” OR “tooth loss” AND "gastric"	0

### Study selection

In this study, the presence of PD was considered whether, at least, one of these clinical characteristics occurs: gingivitis, periodontitis and tooth loss.^5^^,^^20^ This systematic review included case-controls and cohorts studies, and the authors excluded cross-sectional, experimental, animal studies, and case reports. Thus, the authors used the Rayyan software to identify eligible studies and exclude duplicates.^21^ The researchers (FJNA and MAF) retrieved data from studies independently based on titles and abstracts of the eligible studies according to the question of the systematic review (Are patients with periodontal disease at risk for developing gastric adenocarcinoma?). Moreover, references of the selected articles were reviewed to find relevant studies.

### Data extraction

The following variables were collected: (1) First author, year of the publication, and place of the study; (2) Type and period of the study; (3) Sample size, sex, and age; (4) Exposure presence of PD; (5) Diagnosis method for the exposure; (6) Outcome gastric cancer; (7) Diagnosis methods: self-declare and clinical examination; (8) Association measures and 95 % CI (OR, RR, HR); and (9) Adjustment variables according to the articles reviewed: Sex; Smoking; Alcohol; socioeconomic status; Intake of vegetables and fruits; BMI; Regular physical activity among others (Tables 2 and 3). The discrepancies between the two reviewers were solved with the participation of a third evaluator (FSM).

**Table 2:** Characteristics of the case-control studies included in the systematic review.

Author/ Year/ Country	Design/ Study period	Sample/ Sex/ Age	Exposure	Diagnostic Method for Exposure	Outcome	Diagnostic Method for Theutcome	Association measure and 95 % CI	Adjustment variables
Watabe et al./ 1998/ Japan[37]	case-control 1996 to 1997	Cases: 242 *F* = 62 (25. 6 %) *M* = 180 (74. 4 %) Controls: 484 40 - 79 years	Gingivitis Tooth loss	Self-report	Gastric Cancer	Pathology	Gingivitis OR= 1.2 (0.8–1.8) Tooth loss OR=1.7 (1.2–2.4)	–
Hiraki et al./ 2008/ Japan[38]	case-control 2001 to 2005	Cases: 5240 *F* = 2541 (48.5 %) *M* = 2699 (51.5 %) Controls: 10,480 *F* = 5082 (48.5 %) *M* = 5398 (51. 5 %) 20 - 79 years	Tooth loss	Self-report	Gastric Cancer *N* = 702	Hospital record	21 teeth left OR=1.0 (reference) 9–20 teeth remaining OR= 1.0 (0.8–1.3) 1–8 teeth remaining OR= 1.1 (0.8–1.5) 0 teeth remaining OR= 0.9 (0.5–1.4)	It acts; Fri; Smoking; Alcohol; Intake of vegetables and fruits; BMI; Regular physical activity.
Shakeri et al./ 2013/ Iran[39]	case-control 2004 to 2011	Cases: 309 *F* = 83 (26.9 %) *M* = 226 (73.1 %) Controls: 613 *F* = 167 (27.2 %) *M* = 446 (72. 8 %) 40 - 75 years	Tooth loss: Category 1 (≤ 12); Category 2 (13–18); Category 3 (19–24); Category 4 (25–31); Category 5 (32)	Clinical examination	Cardia GC *N* = 1 61 Non-cardia GC *N* = 118	Histology	Tooth loss All gastric adenocarcinomas OR= (reference= Category 1(≤12)) Category 2(13–18) OR=0.5(0.2–1.1) Category 3(19–24) OR =0.9(0.4–1.7) Category 4(25–31) OR =1.6(0.8–3.2) Category 5(32) OR =1.4(0.6–3.0) Cardia GC OR = (reference = Category 1(≤12)) Category 2(13–18) OR = 0.6 (0.2–1.9) Category 3(19–24) OR = 1.6 (0.6–4.35) Category 4(25–31) OR = 3.5 (1.2–9.7) Category 5(32) OR = 1.4 (0.4–4.5) Non-cardia GC OR = (reference= Category 1(≤12)) Category 2(13–18) OR = 0.3 (0.1–1.2) Category 3(19–24) OR = 0.4 (0.1–1.3) Category 4(25–31) OR = 1.7 (0.5–5.6) Category 5(32) OR = 2.1 (0.6–6.9)	It acts; Andthnicity; Education; Consumption of fruits and vegetables, Opium or Tobacco; Denture use. Socioeconomic status

CI, confidence interval; F, female; M, male; CG, gastric cancer; *OR, odds ratio*; RR, relative risk; *HR, hazard ratio*; BMI, body mass index; COPD, chronic obstructive pulmonary disease; ICD, international classification of diseases.

**Table 3:** Characteristics of cohort studies included in the systematic review.

Author/ Year/ Country	Design/ Study period	Sample/ Sex/ Age	Exposure	Diagnostic Method for Exposure	Outcome	Diagnostic Method for Outcome	Association measure and 95 % CI	Adjustment variables
Michaud et al./ 2008/ USA[31]	Prospective 1986 to 2004	48,375 men health professionals 40 - 75 years	Periodontal disease; Tooth loss.	Self-report and imaging	Gastric Cancer *N* = 106	Self-report and Medical records	Periodontal disease In HR*=1.0 (reference) Yes HR*= 1.1 (0.7–1.7) tooth loss (reference=25–32 teeth) HR*= 1.0 17–24 teeth HR*=1.1 (0.6–1.8) 0–16 teeth HR*= 1.1 (0.5–2.1) Periodontal disease In HR**= 1.0 (reference) Yes HR**= 1.3 (0.8–2.0) tooth loss (reference=25–32 teeth) HR**= 1.0 17–24 teeth HR**=1.2 (0.7–2.0) 0–16 teeth HR**= 1.3 (0.6–2.5)	*Smoking history (never; former smoker < 10 years; former smoker >10 years; current smoker 1–14 cigarettes/day; 15–24 cigarettes/day; 25+ cigarettes/day) and packets-year (continuous). **Age (continuous), race (white, Asian and black), physical activity, history of diabetes (yes/no), alcohol (quartile), BMI (<22.22–24.9.25–29.9.30+), geographic location (south, east, northeast, midwest) height (quintiles), calcium intake (quintiles), total caloric intake (quintiles), red meat intake (quintiles), fruit and vegetable intake (quintiles), vitamin D (deciles).
Arora et al./ 2010/ Sweden[43]	Prospective Cohort 1963 to 2004	15. 333 *F* = 8. 433 (55 %) *M* = 6. 900 (45 %) 38 - 77 years	Periodontal disease	Self-report	Gastric Cancer *N* = 193	National Registry	Periodontal disease HR= 0.8 (0.4–1.5) Less mobility HR= 0.8 (0.5–1.3) in disease HR= 1.00 (reference)	Sex (male/female), age (years), education (no schooling/secondary/vocation/other), employment (yes/no/housewife/pensioner/other), number of siblings (ordinal), smoking status (smoker current > 1 pack/day; smoker 〈1 pack/day; former smoker 〉 1 pack/day; former smoker < 1 pack/day; never smoked), partner's smoking status (smoker/ex-smoker/never smoked), smoking status of alcohol (alcohol drinker/ex drinker/never), diabetes (yes/no) and BMI (<20, 20–24.9, 25–29.9.>30 kg/m2).
Wen et al./ 2013/ Taiwan[44]	Retrospective Cohort 1997 to 2010	144.896 *F* = 71,086 (49. 1 %) *M* = 73,810 (50. 9 %) >20 years	Periodontitis (ICD-9: 523.3 and 523.4) and Gingivitis (ICD-9: 523.0 and 523.1)	Positive diagnosis and treated at least 3 times	Gastric Cancer *N* = 151	Histology through national registry	RR= 1.0 (1.0–1.1) Adjusted HR*= 0.9 (0.7–1.1)	*Fri; It acts; Presence of comorbidity.
Chung et al./ 2015/ Taiwan[45]	Retrospective Cohort 2002 to 2009	40.140 *F* = 20,190 (50. 3 %) *M* = 19,950 (49. 7 %) >40 years	chronic periodontitis	Positive diagnosis based on symptoms, medical history and diagnostic test result.	Gastrointestinal cancer (ICD-9: 150–159) *N* = 1084	Medical records	Adjusted HR= 1.2 (1.1–1.2)	Monthly income; Geographic region; Diabetes.
Nwizu et al./ 2017/ USA[30]	Prospective Cohort 1999 to 2013	65,869 post-menopausal women 50 - 79 years	Periodontal disease	Self-report	Stomach	Self-report in biennial questionnaires and medical records	Stomach HR=1.5 (0.9–2.6)	–
Chou et al./ 2018/ Taiwan[46]	Retrospective Cohort 2001 to 2010	50,970 *F* = 24,850 (48.7 %) *M* = 26,120 (51.3 %) 35 - 80 years	Moderate and severe periodontal disease	Database: diagnosed patients who received an additional procedure code for periodontitis.	Stomach (*N* = 101)	Patients diagnosed with gastrointestinal cancer according to the ICD-O-3 (C-16)	*HR=1.0 (0.6–1.4)*	It acts; Fri; Comorbidities (diabetes mellitus/colectomy); index of Charlson; Medication (aspirin/NSAIDs); Socioeconomic level (estimated monthly income and educational level).

CI, confidence interval; F, female; M, male; CG, gastric cancer; OR, odds ratio; RR, relative risk; HR, hazard ratio; BMI, body mass index; COPD, chronic obstructive pulmonary disease; DHF, international classification of diseases; DMFT, decayes, missing and filled teeth.

### Risk of bias

The Newcastle-Ottawa Scale (NOS) was applied to evaluate the quality of selected studies, by two independent, previously trained and approved reviewers. The methodological is divided into three components: group selection (0‒4 points), quality of adjustment for confounding (0‒2 points), and exposure assessment after outcome (0‒3 points). The maximum score can be 9 points, which represents high methodological quality.^22^ A the funnel plot was carried out to assess the risk of publication bias.^23^

### Statistical analysis

For this meta-analysis, the authors considered the following measures of association: Odds Ratio (OR); Relative Risk (RR): Hazard Ratio (HR) and their respective 95 % Confidence Intervals (95 % CI). Accordingly, the authors carried out fixed and random effects models using the “metan” command.^24^ The heterogeneity among the studies was assessed by the I^2^ statistic, where I^2^ = 0‒25 % indicated low heterogeneity; I^2^ = 25 %‒50 %, moderate heterogeneity; and I^2^ > 50 %, high heterogeneity.^25^ The authors used the random effects model in case of high heterogeneity among studies and the software to perform the meta-analysis was STATA 15.

## Results

### Study selection

**Fig. 1:**
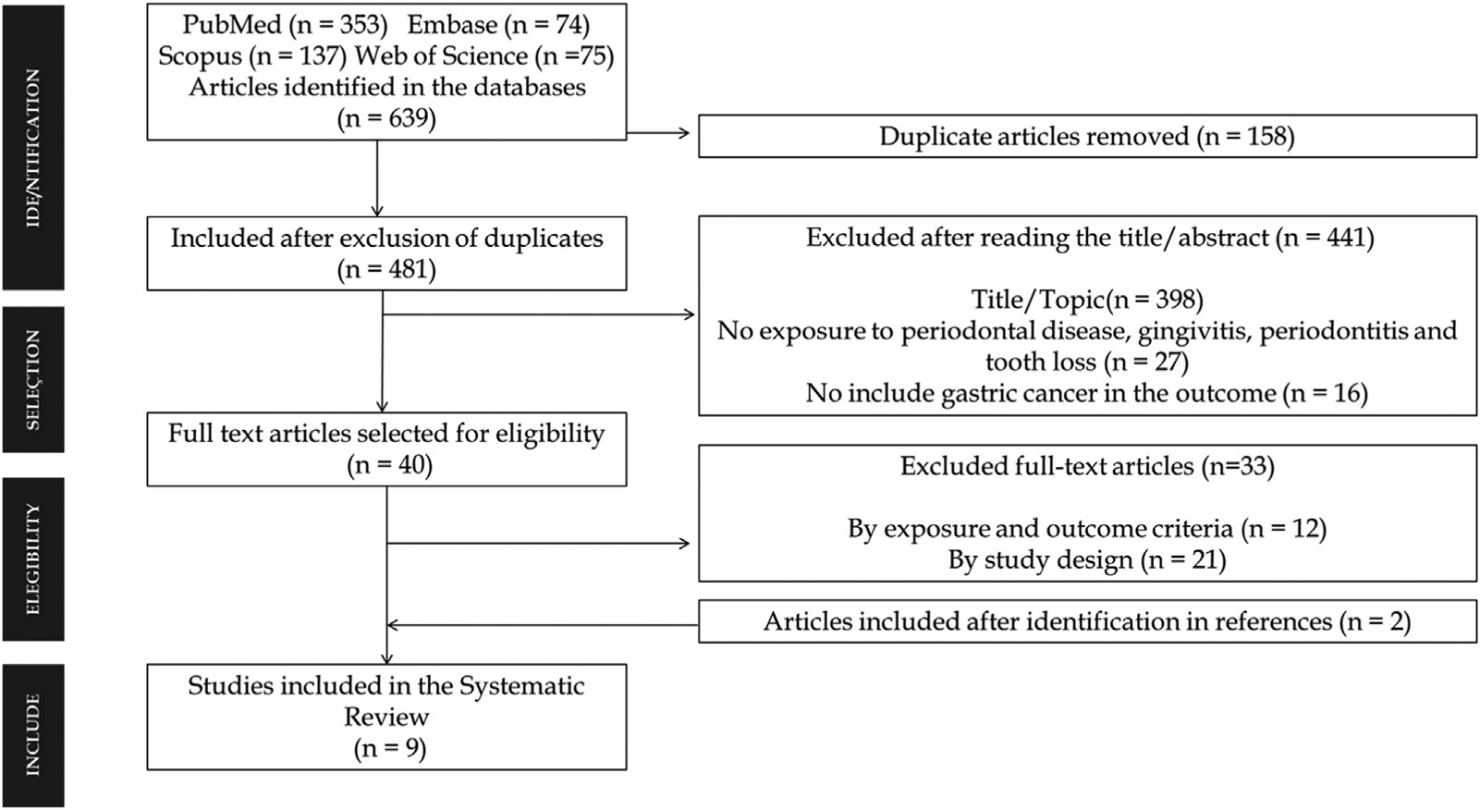
Prisma Selection of eligible studies for the systematic review.

The authors have found 639 articles between 1961 and 2022 (Fig. 1). After reading their title/abstract, the study excluded 441. From that 40 articles were considered eligible for a full reading. Seven studies were shortlisted for the meta-analysis,^16^^,^^26^, ^27^, ^28^, ^29^, ^30^, ^31^ two additional articles were included.^32^^,^^33^ Thus, nine articles were included in this systematic review, three case control studies^26^^,^^27^^,^^32^ and six cohort studies^16^^,^^28^, ^29^, ^30^, ^31^^,^^33^ published between 1998 and 2018. The authors found a population of 2884 cases patients with GAC in the nine studies included.

About six studies were carried out in Asia accounting 2280 gastric adenocarcinoma cases and the diagnostic criteria for PD was the clinical examination (Tables 2 and 3).

**Fig. 2:**
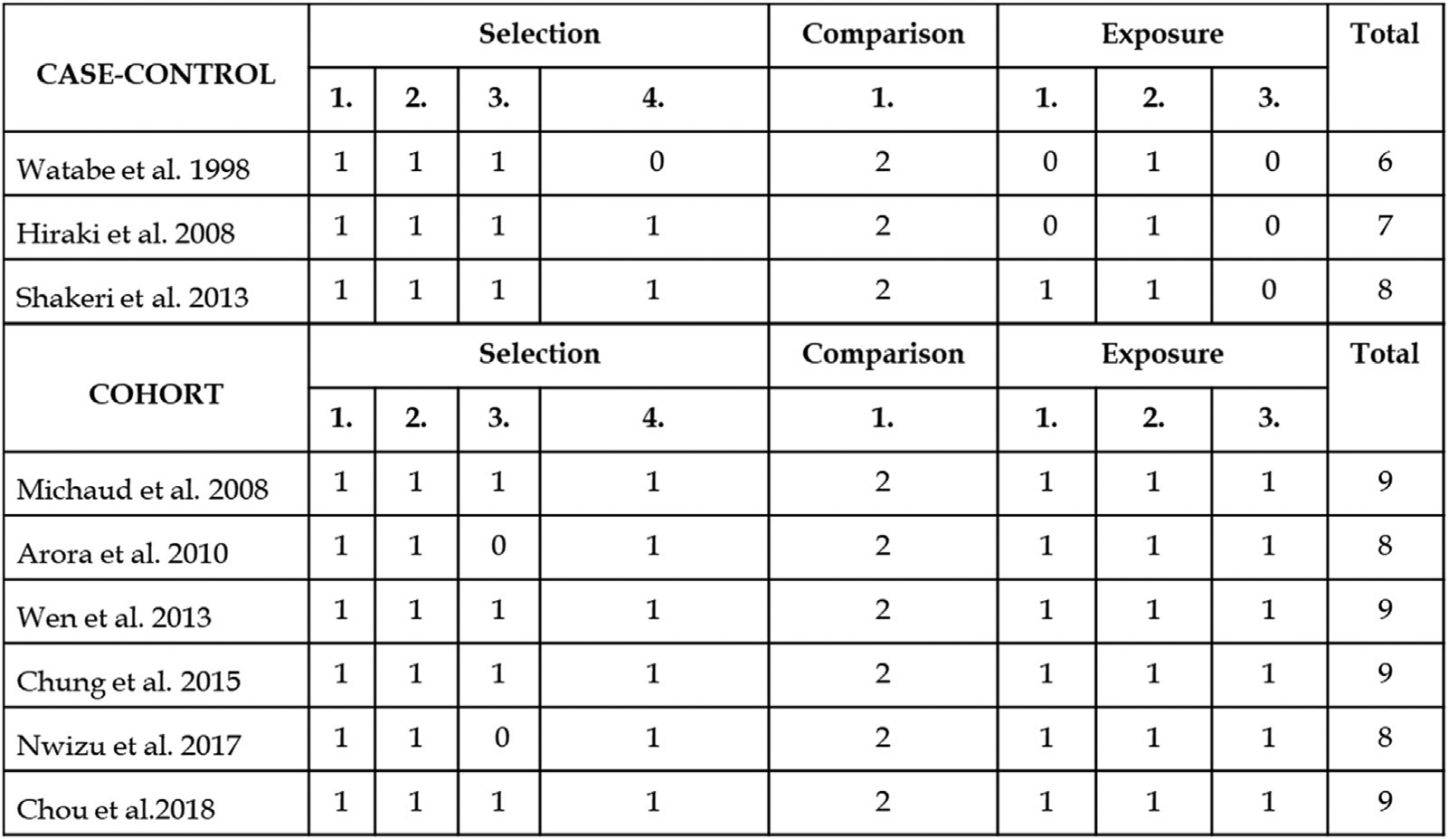
Evaluation of the methodological quality of case-control and cohort studies according to the Newcastle ‒ Ottawa Scale (Wells et al., 2014).

In cohort studies, the NOS ranged from eight points^30^^,^^33^ to nine points,^16^^,^^28^^,^^29^^,^^31^ and between six and eight points^26^^,^^27^^,^^32^ among case-control studies selected therefore selected studies have high scores in quality (Fig. 2).

In cohort studies, the NOS ranged from eight points^30^^,^^33^ to nine points.

### Summary of meta-analysis

**Fig. 3:**
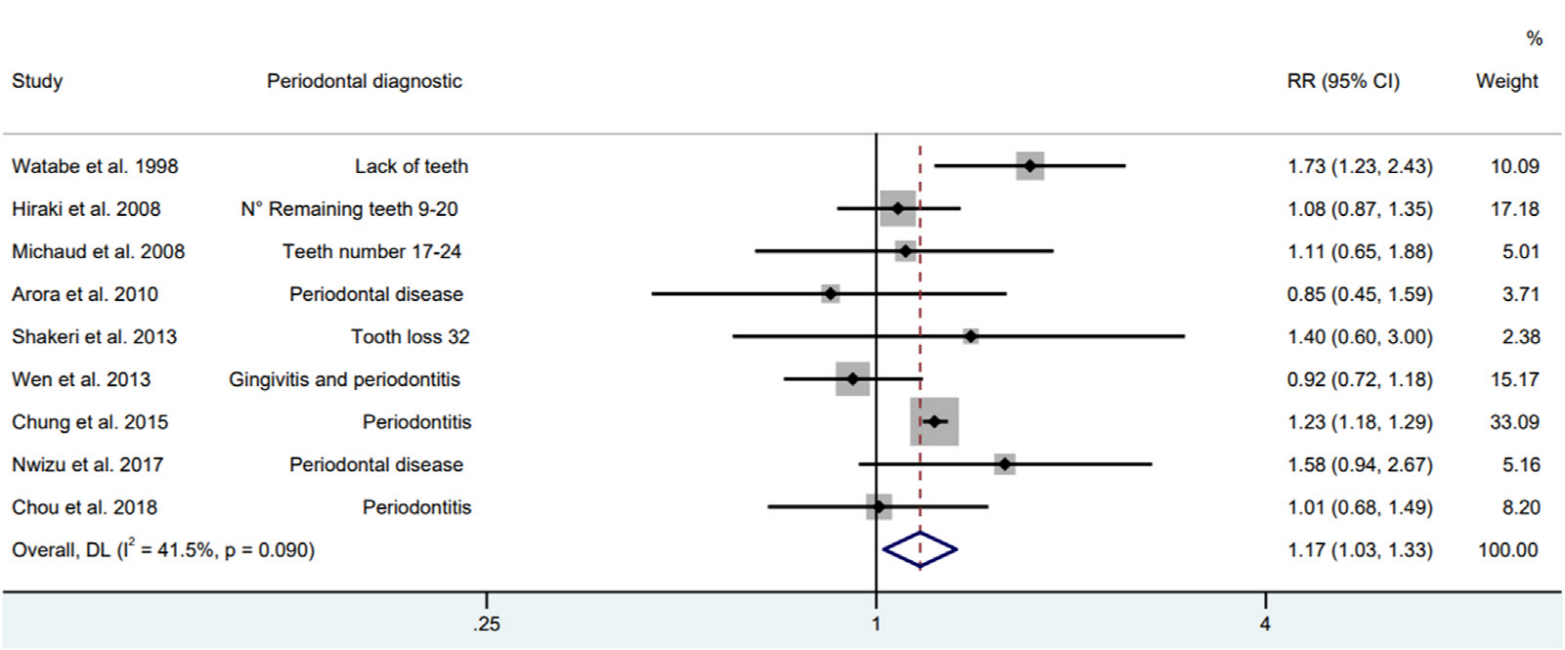
Forest plot of cohort and case-control studies between periodontal disease and gastric adenocarcinoma (Random model).

**Fig. 4:**
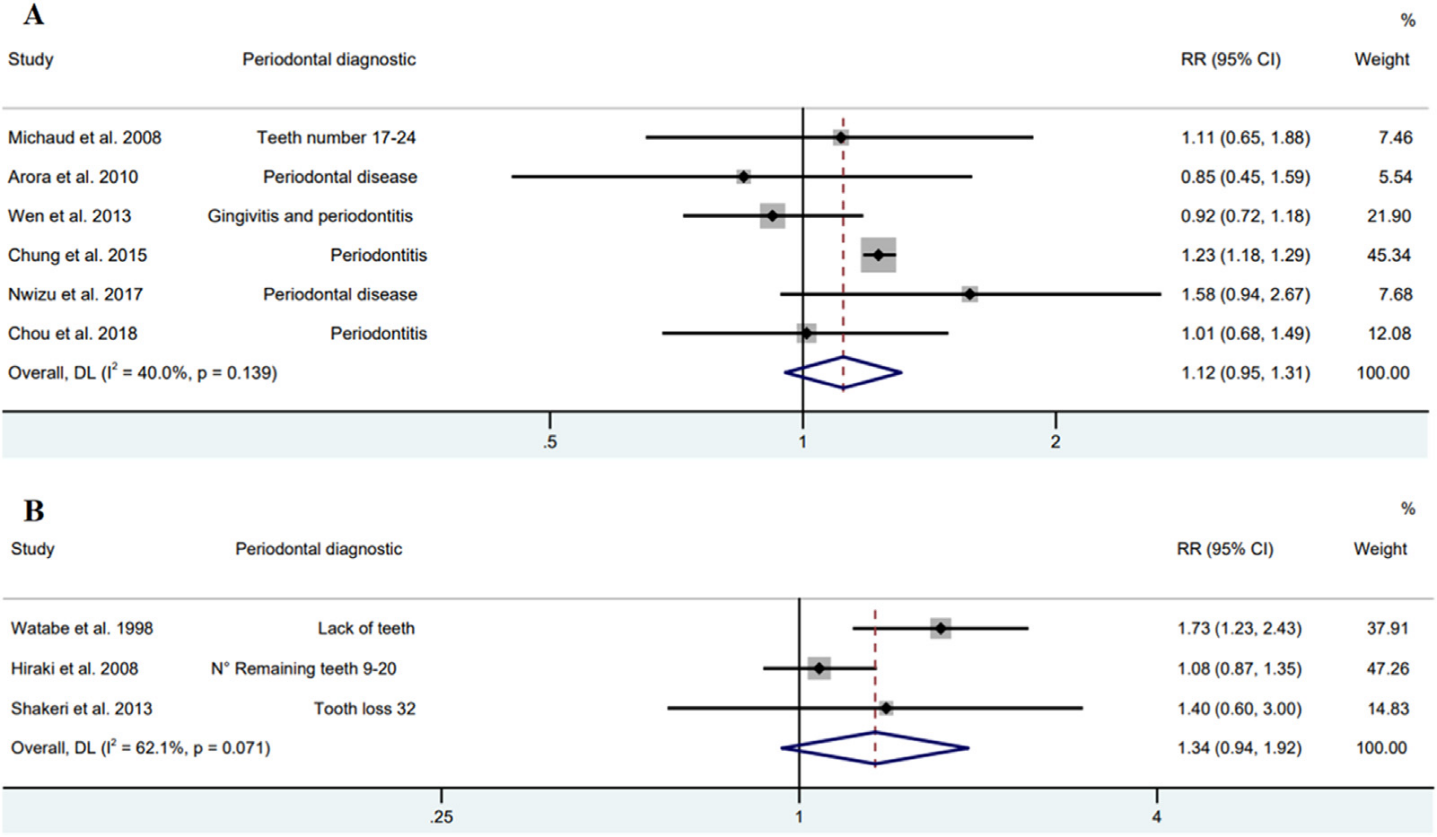
Forest plot of cohort (A) and case-control (B) studies between periodontal diseases and gastric adenocarcinoma (Random model).

In the meta-analysis of nine observational studies, the presence of PD was associated to an increase in the risk of GAC by 17 % (RR = 1.17; 95 % CI 1.03‒1.32), with a heterogeneity of 39.9 % (Fig. 3). While in the subgroup analysis, cohort and case-control studies had no association with PD and GAC (Fig. 4).

**Fig. 5:**
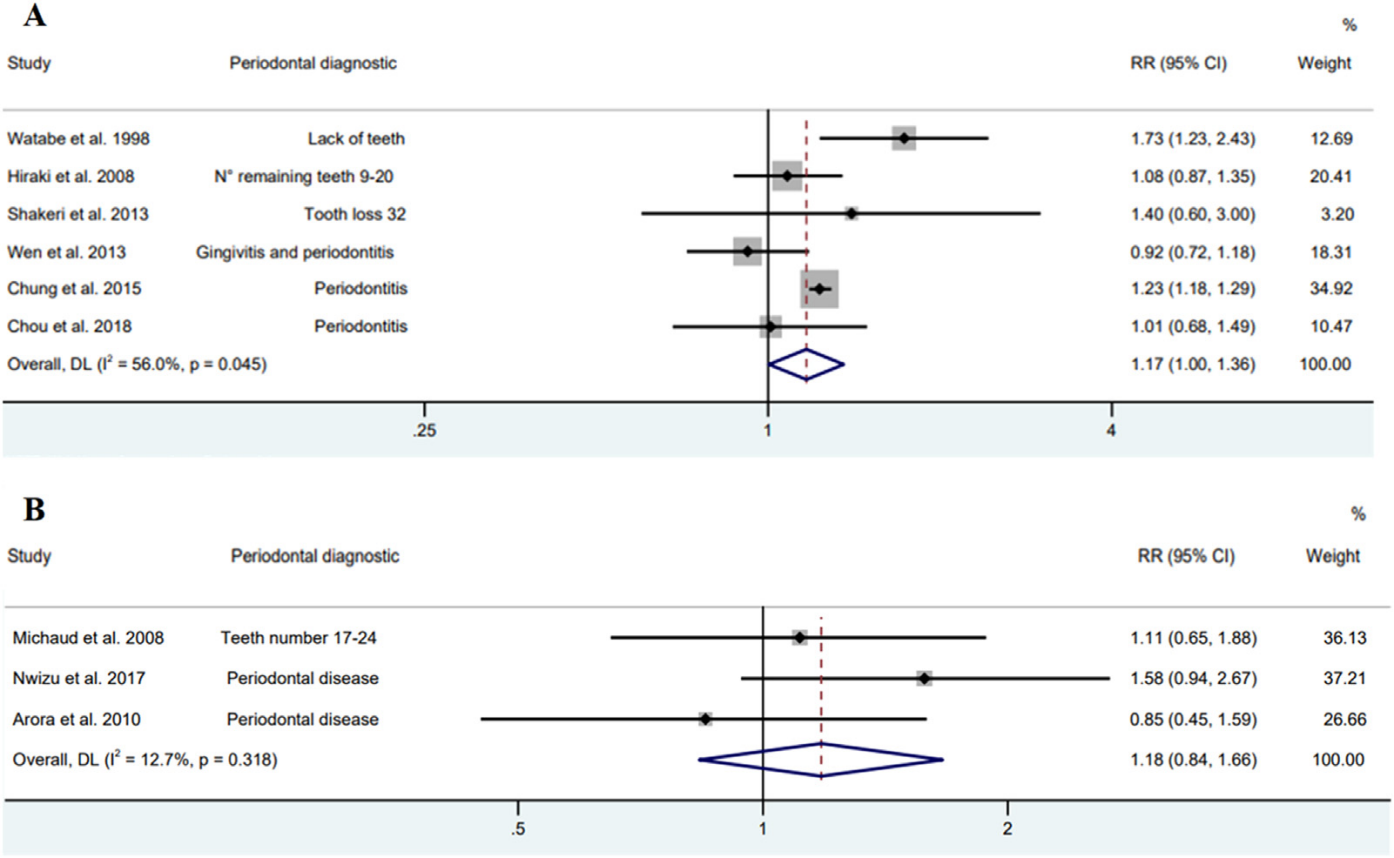
Forest plot of Asian (A) and American and European (B) studies between periodontal diseases and gastric adenocarcinoma (Random model).

An association between GAC and PD was found to be a risk in the Asian population (RR = 1.17; 95 % CI 1.00‒1.36); however, in American and European studies there was no risk (RR = 1.18; 95 % CI 0.84‒1.66) (Fig. 5).

**Fig. 6:**
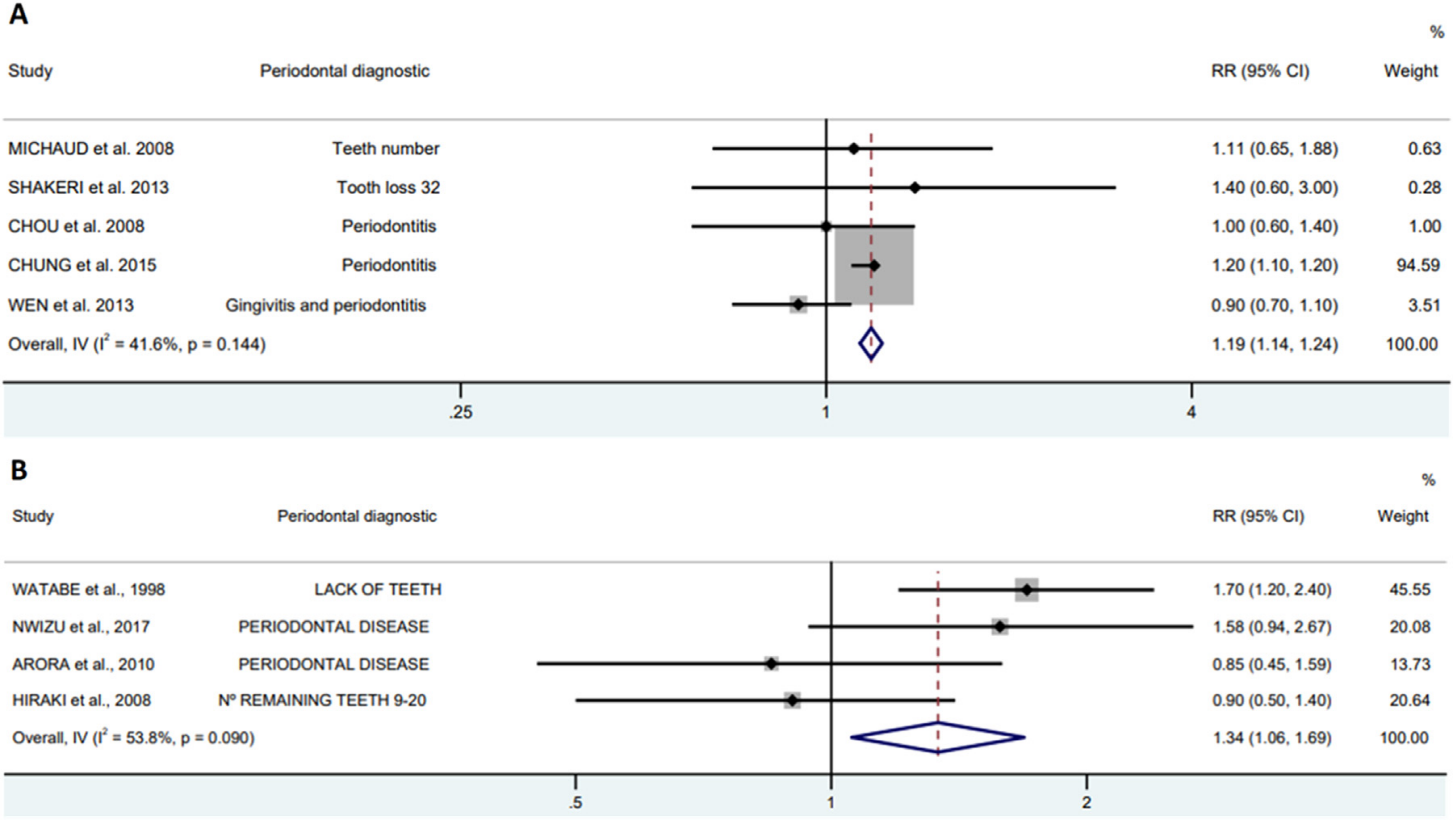
Forest plot of periodontal diagnostic, clinical examination (A) self-report (B) between periodontal diseases and gastric adenocarcinoma.

To evaluate the presence of PD In epidemiological studies, it can be assessed by patient self-report and by clinical examination. The authors observed an increased risk of patients with PD presenting GAC, that remained regardless of the diagnostic method for PD, 19 % (RR=1.19; 95 % CI 1.14‒1.24) to 34 % (RR = 1.34; 95 % CI 1.06‒1.69) for clinical examination and self-report, respectively (Fig. 6).

**Fig. 7:**
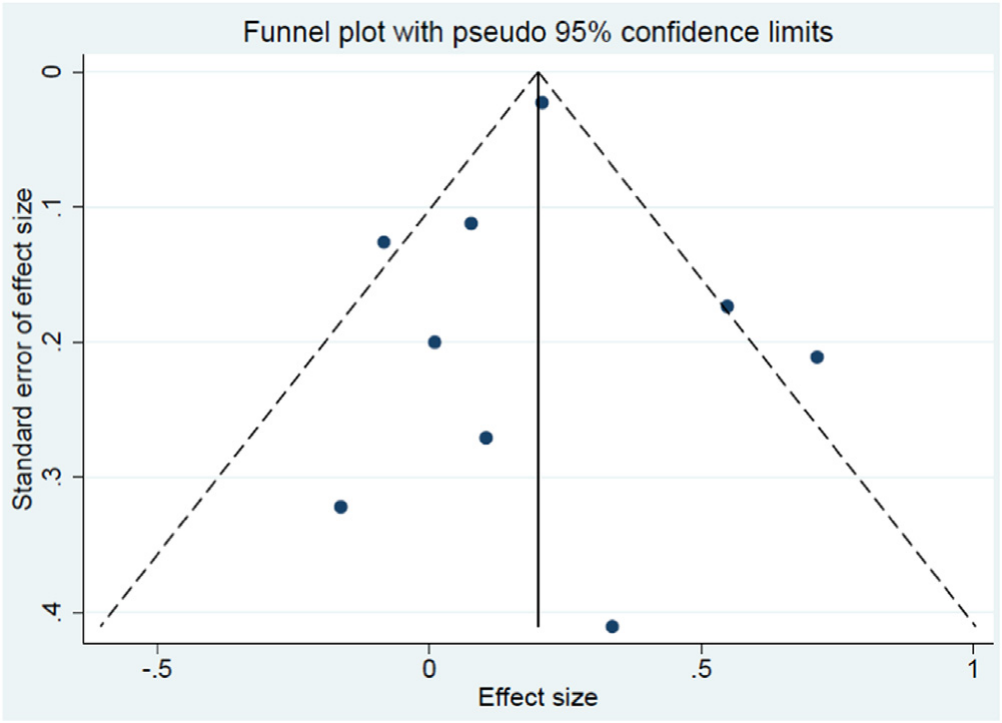
Funnel plot of all studies included in the meta-analysis.

No publication bias was observed in the selected studies according to the Egger test (*p* = 0.860) (Fig. 7).

## Discussion

To our knowledge, this is the first meta-analysis to explore the association of PD with the risk of GAC. In this study, patients with PD were at risk of developing GAC, which validates the hypothesis that PD is proposed as a potential carcinogenic factor.^16^ The authors also observed that the risk for GAC continues regardless of the diagnostic method for PD. However, there were differences between populations; Asian patients were at risk of developing GAC associated with PD than Americans and Europeans. It is necessary to point out that the studies had several adjustment variables: Sex; Smoking; Alcohol; socioeconomic status; Intake of vegetables and fruits; BMI; Regular physical activity. Therefore, the present results highlight that periodontal diseases have a significant effect on GAC, further studies are needed to assess how this mechanism occurs and which other microorganisms may be linked to oral-gastric dysbiosis.

Gastric cancer is the leading cause of death among men in South Asian countries.^1^ This study found an association between periodontal disease and gastric adenocarcinoma in Asian studies, as opposed to American and European studies. Asian populations have polymorphisms of the interleukin genes (IL-17 and IL-10) that increase the risk of gastric cancer, due to their interaction with *H. pylori* and the habit of smoking.^34^ These same genetic polymorphisms can cause phenotypic differences in the inflammatory responses in PD, which are important in the individual's sensitivity to the disease, in the progression of the disease or in the response to treatment.^35^ The prevalence of PD varies between 16 % (Western Pacific region) and 23 % (Africa region), while case numbers reflect the demographic share of the respective regions, with Southeast Asia and Western Pacific regions having the highest number of cases and the Eastern Mediterranean region with the lowest number of PD cases.^4^

The gold standard for evaluating PD is probing all teeth and radiographic interpretation.^36^^,^^37^ However, in studies conducted with large populations, they are less feasible since they require a big number of trained examiners, high-costly dental equipment, and infection control protocols that demand unpractical execution time.^37^, ^38^, ^39^ Self-report and clinical examination are an accessible, reliable, and cost-saving method.^39^, ^40^, ^41^ In this meta-analysis, the authors observed an increased risk of PD patients developing GAC, regardless of the diagnostic method used for PD.

This meta-analysis presents limitations. There is a missing information among the studies, such as sex^16^^,^^26^^,^^27^^,^^30^, ^31^, ^32^, ^33^ topography (cardia and non-cardia),^16^^,^^26^^,^^30^, ^31^, ^32^, ^33^ and different age groups.^16^^,^^26^^,^^30^, ^31^, ^32^, ^33^ In addition to what studies use as a proxy for PD (gingivitis, periodontitis and tooth loss), therefore, the authors included it in the systematic review. However, it has strength as many participants providing accurate risk estimates, and a high methodological quality of the selected studies. Furthermore, the sensitivity analysis identified that patients with PD may be associated with the development of GAC, regardless of the diagnostic method for PD.

Since there is not enough evidence demonstrating how this association between PD and risk of GAC occurs. Additional studies with more detailed PD data and assessment of the oral microbiome may provide more clarity.

## Conclusion

The presence of PD increased the risk of GAC. Several studies suggest that the infectious-inflammatory process of PD can initiate complex reactions involving inflammation mediators and microorganisms that may link the risk of tumor development, therefore there are biological bases to support a relationship between PD and GAC, but more studies are needed to assess the depth of this connection. In addition to considering the screening of patients at potential risk for GAC in the clinical practice of dentists.

### PRISMA 2009 checklist statement

The authors have read the PRISMA 2009 Checklist, and the manuscript was prepared and revised according to it.

## Conflicts of Interest

The authors declare no conflicts of interest.
